# Zero fluoroscopy catheter ablation for atrial fibrillation: a systematic review and meta-analysis

**DOI:** 10.3389/fcvm.2023.1178783

**Published:** 2023-06-16

**Authors:** Dorottya Debreceni, Kristof Janosi, Botond Bocz, Marton Turcsan, Reka Lukacs, Tamas Simor, Bor Antolič, Mate Vamos, Andras Komocsi, Peter Kupo

**Affiliations:** ^1^ Heart Institute, Medical School, University of Pecs, Pecs, Hungary; ^2^ Department of Cardiology, University Medical Center Ljubljana, Ljubljana, Slovenia; ^3^ Cardiac Electrophysiology Division, Department of Internal Medicine, University of Szeged, Szeged, Hungary

**Keywords:** zero fluoroscopy, meta-analysis, atrial fibrillation, catheter ablation, pulmonary vein isolation

## Abstract

**Introduction:**

Catheter ablation for atrial fibrillation (AF) is the most frequently performed cardiac ablation procedure worldwide. The majority of ablations can now be performed safely with minimal radiation exposure or even without the use of fluoroscopy, thanks to advances in 3-dimensional electroanatomical mapping systems and/or intracardiac echocardiography. The aim of this study was to conduct a meta-analysis to compare the effectiveness of zero fluoroscopy (ZF) versus non-zero fluoroscopy (NZF) strategies for AF ablation procedures.

**Methods:**

Electronic databases were searched and systematically reviewed for studies comparing procedural parameters and outcomes of ZF vs. NZF approaches in patients undergoing catheter ablation for AF. We used a random-effects model to derive the mean difference (MD) and risk ratios (RR) with a 95% confidence interval (CI).

**Results:**

Our meta-analysis included seven studies comprising 1,593 patients. The ZF approach was found to be feasible in 95.1% of patients. Compared to the NZF approach, the ZF approach significantly reduced procedure time [mean difference (MD): −9.11 min (95% CI: −12.93 to −5.30 min; *p* < 0.01)], fluoroscopy time [MD: −5.21 min (95% CI: −5.51 to −4.91 min; *p* < 0.01)], and fluoroscopy dose [MD: −3.96 mGy (95% CI: −4.27 to −3.64; *p* < 0.01)]. However, there was no significant difference between the two groups in terms of total ablation time [MD: −104.26 s (95% CI: −183.37 to −25.14; *p* = 0.12)]. Furthermore, there was no significant difference in the acute [risk ratio (RR): 1.01, 95% CI: 1.00–1.02; *p* = 0.72] and long-term success rates (RR: 0.96, 95% CI: 0.90–1.03; *p* = 0.56) between the ZF and NZF methods. The complication rate was 2.76% in the entire study population and did not differ between the groups (RR: 0.94, 95% CI: 0.41–2.15; *p* = 0.89).

**Conclusion:**

The ZF approach is a feasible method for AF ablation procedures. It significantly reduces procedure time and radiation exposure without compromising the acute and long-term success rates or complication rates.

## Introduction

Atrial fibrillation (AF) is the most prevalent sustained cardiac arrhythmia, which is linked to an elevated risk of stroke, heart failure, mortality, and reduced quality of life (1). The electrical isolation of the pulmonary veins is the cornerstone of AF ablation procedures for patients with symptomatic paroxysmal or persistent AF that is refractory to antiarrhythmic drug (AAD) therapy (2). Catheter ablation for AF is by far the most commonly performed cardiac ablation procedure worldwide (2–4).

Radiation exposure during electrophysiology (EP) procedures can vary significantly in clinical practice. During AF ablation procedures, the average fluoroscopy exposure is 15 mSv, which is higher compared to other ablations and carries an excess risk of fatal and non-fatal cancer of 1 in 750 men at the age of 50 years (5, 6). Moreover, based on the stochastic effects of the radiation, there is no safe lower threshold. Thus, completely fluoroless procedures can entirely eliminate radiation hazards for both patients and personnel, although radiation risk can be reduced with minimal fluoroscopic approach also.

Due to the technological progress made in the last decade, with the use of 3-dimensional electroanatomical mapping systems (EAMS) and/or intracardiac echocardiography (ICE), the majority of the ablations can be performed safely with minimal radiation exposure or even without the use of fluoroscopy (7–10).

Low or zero (L/Z) fluoroscopy catheter ablation are available also for pulmonary vein isolation (PVI) procedures. A previous meta-analysis published in 2020 included 2,228 patients who underwent AF ablation, L/Z fluoroscopy-guided approaches were compared to conventional, fluoroscopy-guided procedures. The L/Z fluoroscopy approach was associated with shorter procedural time and reduced fluoroscopy exposure, without compromising safety or efficacy compared to traditional AF ablation techniques (11).

Nonetheless, there is limited available scientific data regarding completely fluoroless AF ablation procedures. Thus, we conducted a systematic review and a meta-analysis to analyse the feasibility, safety and efficacy of zero-fluoroscopy approach for AF ablation procedures.

## Methods

### Search strategy and data acquisition

Electronic databases [PubMed, Excerpta Medica Database (EMBASE), Cochrane Central Register of Controlled Trials (CENTRAL)] were systematically searched for relevant articles published between January of 2000 and December of 2022, using the search string “zero-fluoroscopy or fluoroless or non-fluoroscopic” and “ablation” and “atrial fibrillation”. Additionally, manual searches of reference lists of relevant studies were conducted to identify any additional articles that were not found in the database search. Reviews and duplicate articles were excluded. The analyses were performed in accordance with the Preferred Reporting Items for Systematic Review and Meta-Analyses (PRISMA) guidelines.

In this meta-analysis, we included studies that fulfilled the following criteria:

(1) Randomized or non-randomized prospective and retrospective studies enrolling consecutive patients with paroxysmal or persistent AF who underwent catheter ablation for AF; (2) studies having at least 1 zero-fluoroscopic (ZF) and 1 non-zero-fluoroscopic arm (NZF); (3) studies written in English. Case reports, letters, conference abstracts and presentations as well as full text papers not in the English language were excluded. ZF was defined as no radiation used during the procedure. “Low fluoroscopic” and “minimal fluoroscopic” procedures were not considered as ZF approach. All approaches other than ZF were considered NZF. Selection and data abstraction were done independently by two reviewers (DD and PK) and any disagreements were resolved by consensus.

We extracted the following data from the included studies: the first author's name, publication year, study design, number of patients in each group, baseline characteristics of the study population, as well as procedural and clinical outcome data for our meta-analysis.

### Endpoints of interest

The primary endpoints of the study were the skin-to-skin procedure time and any procedure related complications, including vascular complications (groin hematoma, pseudoaneurysm, arteriovenous fistula), cardiac effusion/tamponade, stroke/cardioembolic events, phrenic nerve palsy and death. The secondary outcomes were fluoroscopy exposure, total ablation time, and acute and long-term success rates.

### Statistical analysis

We performed the analyses in R statistical software package version 4.2.2 (R Development Core Team, 2010) with the help of the “dmetar” package (12). A random-effects model was used to derive risk ratios (RR) with 95% confidence interval (CI) on dichotomous outcomes and mean difference (MD) on continuous data. The significance of the pooled estimates was determined by the *Z*-test, and *p* < 0.05 was considered as statistically significant. Heterogeneity was tested with a chi-square heterogeneity statistic for which a *p* value <0.2 was considered potentially heterogeneous. Consistency was assessed by the *I*^2^ statistic, which describes the percentage of total variation across studies that is due to heterogeneity rather than due to chance. Values of *I*^2^ < 25% were considered as low and values of *I*^2^ > 75% were considered as high. To assess the stability of acquired effect estimates, a leave-one-out sensitivity analysis was performed. Quality assessment was performed with Cochrane's tool for assessing bias, wherein studies are scored as high, low, or unclear risk of bias in five domains: selection, performance, detection, attrition, and reporting. Funnel plot was drawn to assess publication bias, and asymmetry was assessed by visual estimation and by Egger's linear regression test.

## Results

### Study characteristics

**Figure 1:**
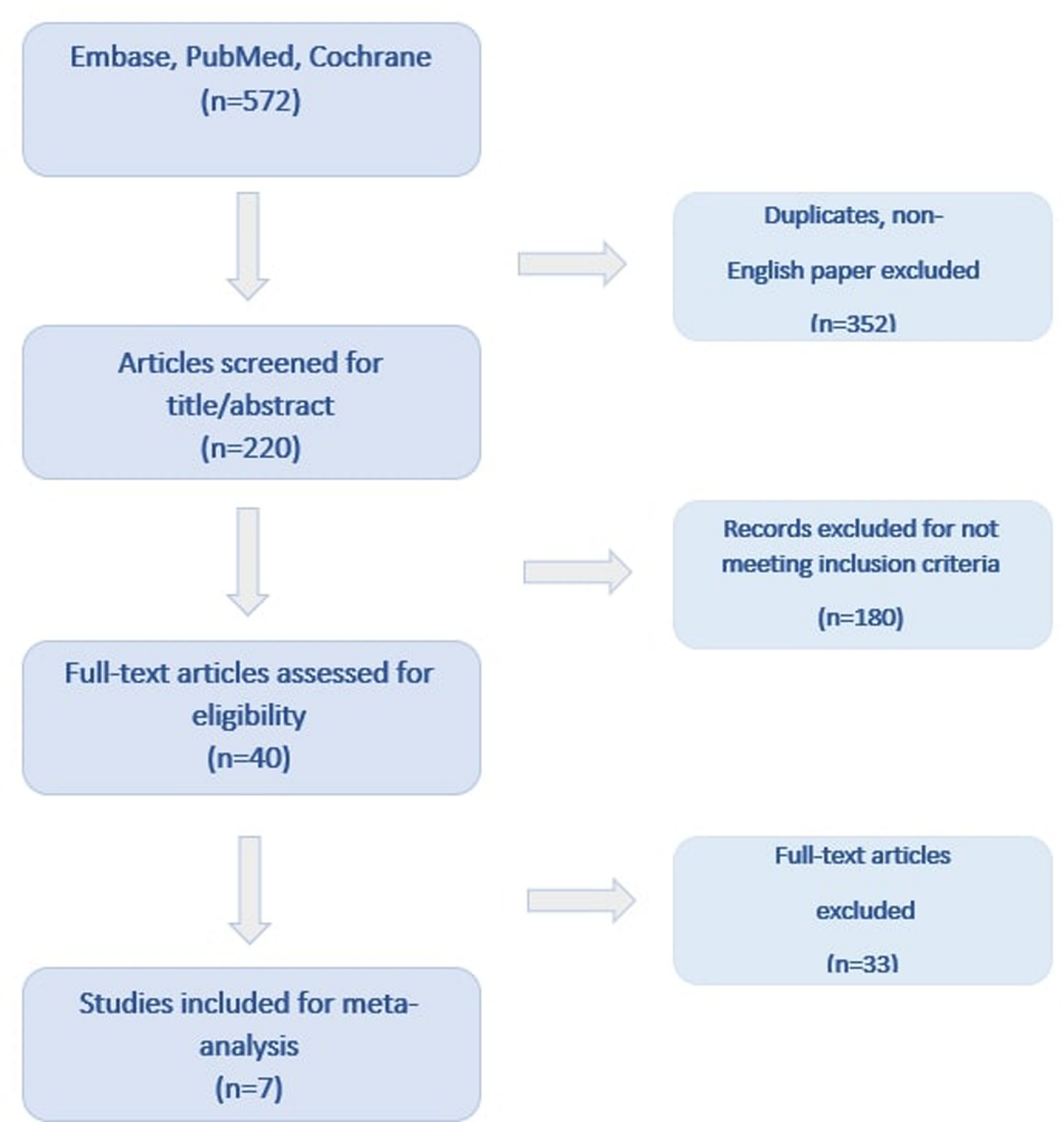
Flowchart of study selection.

Seven studies involving 1,593 patients (726 patients in the ZF and 867 patients in the NZF group) included in our analysis. Among the included studies, 1 was a randomized controlled trial (RCT) (13) and 6 were observational, non-randomized (14–19). The results of the literature search are presented in Figure 1 and the main characteristics of the trials and study populations are summarized in Table 1. Except for 1 trial that enrolled patients with patent foramen ovale (PFO) (18), ICE was also applied to achieve the ZF strategy in addition to EAMSs. In 1 trial, AF ablation procedures were performed using CARTO, Ensite Precision and Rhythmia EAMSs (15), while in the other studies, CARTO 3 system was exclusively used. The ZF approach was feasible in 95.1% of the patients, and in remaining cases, fluoroscopy was used. The mean length of the follow-up period varied between 3 and 15.2 months.

**Table 1:** Study and patients’ characteristics of the included trials.

First author	Year	Design	Patient number	Number of ZF patients	AF type	Operators	Operators’ experience with ZF	EAM system	Use of ICE	Additional line/+ procedures (number of patients in ZF/NZF)	Feasibility of zero PVI (%)	Mean follow-up (months)	Sex (male/female number)	Mean age (ZF/NZF)	Mean BMI or weight (ZF/NZF)	LV ejection fraction (%) (ZF/NZF)	LA diameter (mm) (ZF/NZF)
Bulava	2015	Randomized, controlled trial	80	40	Paroxysmal	3 (ZF: 1)	NA	CARTO	Yes	CTI, LA lines (10/7)	98%	12	52/28	61.6 ± 9.9 60.2 ± 11.1	29.7 ± 8.4 28.3 ± 3.5	66.6 ± 6 65 ± 7	43.0 ± 5.7;42.2 ± 7.2
Gul	2021	Retrospective, non-randomized	247	116	Paroxysmal, persistent	1	Yes	CARTO	Yes	LA roof line, LA posterior wall, mitral isthmus (1/10)	NA	12	150/97	61.9 ± 10.6 62.6 ± 10.6	29.8 ± 6.2 29.65 ± 4.6	NA	41.6 ± 5.5;41.5 ± 5.2
Lurie	2021	Retrospective, non-randomized	323	147	Paroxysmal, persistent	3	No	CARTO, Ensite, Rhythmia	Yes	lines, CTI (82/67)	88.5%	12	216/107	59.2 ± 10 59.7 ± 9.9	30.7 ± 5.3 29.5 ± 4.9	56 ± 7 55 ± 7	42 ± 7 41 ± 8
Lyan	2018	Retrospective, non-randomized	481	245	Paroxysmal	NA	NA	CARTO	Yes	CTI (40/45)	NA	15.2 ± 4.1	254/227	59.7 ± 11.3 60.8 ± 10.6	29.0 ± 4.5 29.1 ± 4.7	62 ± 7.1 61.1 ± 6.8	42.0 ± 4.5;42.0 ± 4.1
Percell	2016	Retrospective, non-randomized	50	20	Paroxysmal, persistent	1	NA	CARTO	Yes	CTI (11/11)	95%	3	28/22	57.8 61.5	NA	53 52	43 42
Scaglione	2020	Prospective, multicenter	250	58	Paroxysmal	NA	NA	CARTO	No	NA	100%	12	197/53	59.7 ± 11.2 59.0 ± 10.5	25.1 ± 3.8 24.5 ± 4.1	59.7 ± 7.3 61.3 ± 6.5	85.1 ± 29.3 89.9 ± 28.0
Zei	2020	Prospective, multicenter	162	100	Paroxysmal, persistent	6	Yes	CARTO	YES	CTI, LA roof line, LA septal line, mitral isthmus line (50/54)	100%	12	125/37	62 ± 8.4 63 ± 8.9	NA	58 ± 9 57 ± 7	44 ± 9 42 ± 7

AF, atrial fibrillation; BMI, body mass index; CTI, cavotricuspidal isthmus, EAM, electroanatomic mapping; ICE, intracardiac echocardiography; LA, left atrium, LV, left ventricular, NA, not available; NZF, non-zero fluoroscopy; PVI, pulmonary vein isolation, ZF, zero fluoroscopy.

### Procedural and outcome data

**Figure 2:**
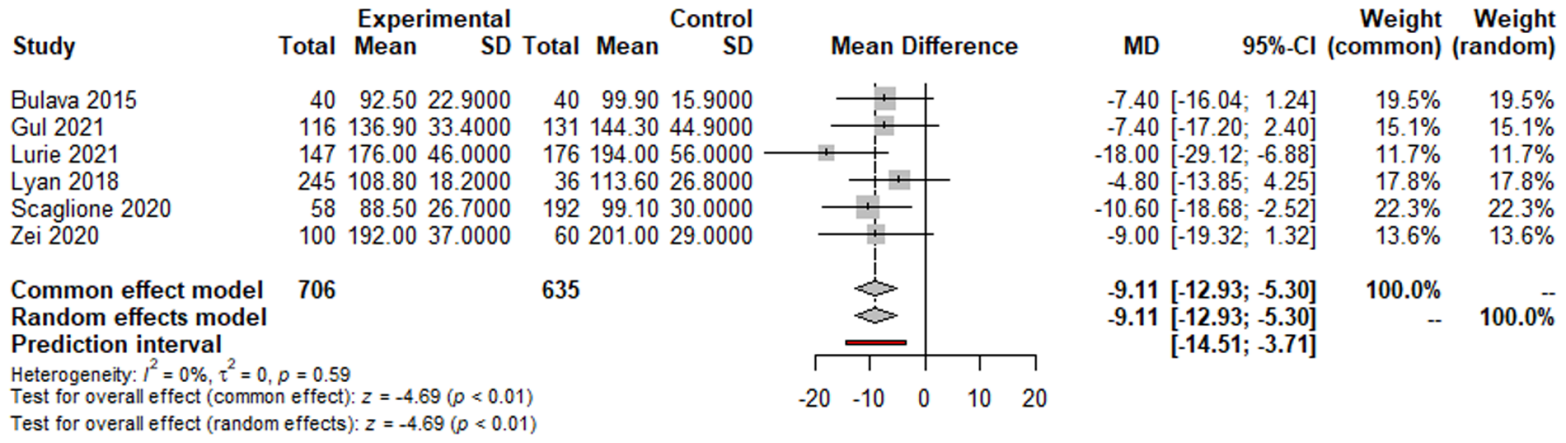
Forest plots of procedure time.

The ZF approach was associated with a significant decrease in procedural procedure time compared to the NZF approach [MD: −9.11 min (95% CI: −12.93 to −5.30 min; *p* < 0.01; Figure 2)]. Additionally, the ZF group had reduced fluoroscopy time [MD: −10.02 min (95% CI, −18.67 to −1.37 min; *p* = 0.02)] and fluoroscopy dose [MD: −3.96 mGy (−4.27 to −3.64 mGy; *p* < 0.01)]; however, the total ablation time was similar [MD: −138.90 s (95% CI: −316.27 to 38.48 s; *p* = 0.12)]. The acute success rate was 99.35% and did not differ between the groups (RR = 1.00, 95% CI, 0.99–1.01; *p* = 0.71). No difference in long-term success rate was found between the groups (RR: 0.98, 95% CI, 0.90–1.06; *p* = 0.56).

**Figure 3:**
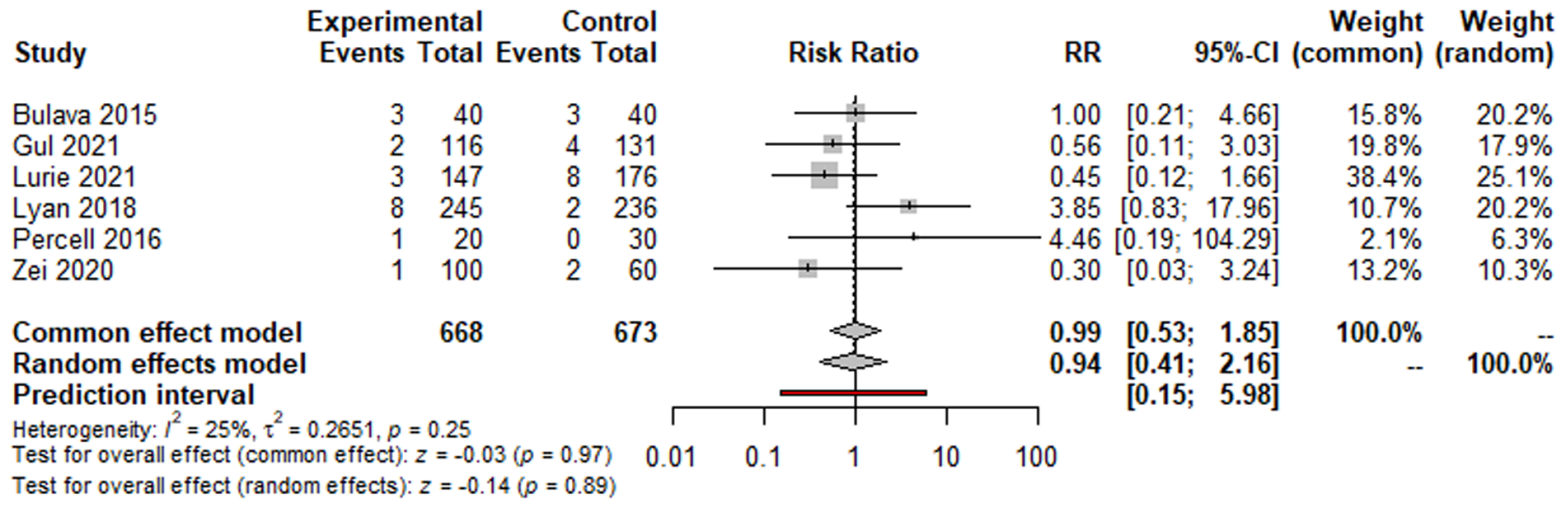
Forest plots of complications.

Regarding safety outcomes, there were 18 cases of complications in the ZF arm (2.69%) and 19 cases in the NZF arm (2.82%). The risk of complications was not significantly different between the two study arms (RR: 0.94, 95% CI: 0.41–2.15; *p* = 0.89; Figure 3). The results for secondary outcomes are summarized in Table 2 and Supplementary Figure S1.

**Table 2:** Summary of outcomes of secondary endpoints.

Outcome	Number of studies	Number of patients	Mean difference (95% CI)	Test for overall effect	Heterogeneity
Total ablation time	4	1,134	−104.26 s (−183.37; −25.14)	*p* = 0.12	*I*^2^ = 72%; *p* = 0.01
Fluoroscopy time	5	1,294	−5.21 min (−5.51; −4.91)	*p* < 0.01	*I*^2^ = 100%; *p* < 0.01
Fluoroscopy dose	5	1,094	−3.96 mGy (−4.27; −3.64)	*p* < 0.01	*I*^2^ = 99%; *p* < 0.01
Acute success rate	6	1,541	1.01 (1.00; 1.02)^a^	*p* = 0.72	*I*^2^ = 11%; *p* = 0.34
Long term success rate	7	1,541	0.96 (0.90; 1.03)^a^	*p* = 0.56	*I*^2^ = 32%; *p* = 0.18

CI, confidence interval.

^a^ Risk ratio (95% CI).

A leave-one-out analysis indicated no difference between the groups for primary outcomes (Supplementary Figure S2). Furthermore, funnel plot analyses revealed no sign of possible publication bias (Supplementary Figure S3).

## Discussion

In this meta-analysis of 7 studies involving 1,593 patients who underwent AF ablation procedures, we found that the use of ZF ablation was associated with a significant reduction in procedural and fluoroscopy time compared to the NZF approach, while maintaining similar efficacy and safety outcomes.

Medical radiation exposure is the most significant anthropogenic source of radiation (20). During EP procedures, fluoroscopy is primarily used for catheter placement and accounts for 95% of the total fluoroscopy time (21). The amount of radiation exposure varies among different types of ablation procedures, with AF ablation procedures associated with the highest doses. These procedures expose patients up to an average dose of 15 mSv per procedure, equivalent to 750 chest x-rays (21). Fluoroscopy increases the life-time risk of cataract, dermatitis, and cancer via stochastic and deterministic effects, thus preventing potentially life-threatening effects of ionizing radiation. For example, a typical PVI procedure raises the absolute lifetime of fatal cancer risk by 0.08% (21). Therefore, radiation exposure must be minimized according to the ALARA principle, which aims to reduce exposure “as low as reasonably achievable” (22).

In the last two decades, technology has significantly improved, and nowadays, EAMSs offer reliable alternatives to fluoroscopy for visualizing catheter positions during EP procedures. By using EAMSs, radiation exposure can be substantially reduced, and completely fluoroless ablations have become available (23). Ensite NavX (St. Jude Medical, Inc., St. Paul, MN, USA) system based on impedance measurements between catheter electrodes and patches put on patient's chest and abdomen, CARTO 3 system (Biosense Webster, Inc., Diamond Bar, CA, USA) uses magnetic location. Some studies showed different results comparing EAMSs (24). In our meta-analysis only CARTO system was used 6 of 7 studies as a support system for PVI. In recent years new ablation techniques were successfully developed for PVI such as ablation index, high-power short- duration, or pulse field ablation, which methods lead differences in procedure time and required fluoroscopy time (25–27). In our meta-analysis, ablation index was used in 1 publication (14) and contact force was available in 6 study (13, 15–19), based on findings during EPS additional lesion delivery was performed (Table 1). The ZF approach was initially used for ablations of supraventricular tachycardias (SVTs) (28). A recent meta-analysis compared Z/MF versus conventional, fluoroscopy-guided techniques for SVT ablations and found that the Z/MF approach reduces radiation exposure and ablation time without compromising the acute and long-term success rates or increasing the complication rate (28).

Fluoroscopic guidance is currently considered the standard method for transseptal puncture (TSP), which remains a major obstacle for widespread adoption of zero-fluoroscopy AF ablation, despite the availability of both transesophageal and intracardiac echocardiographic techniques for achieving fluoroless TSP (23, 29, 30). In addition, the use of intracardiac echocardiography (ICE) has been shown to reduce fluoroscopy exposure during both SVT and AF ablation procedures (7, 31). In our analysis, except for 1 study that enrolled patients with PFO, ICE was used in ZF arms. Besides ICE, recently developed steerable sheaths that can be visualized using EAMSs have also been shown to contribute to the reduction of fluoroscopy exposure and the performance of fluoroless procedures (32).

A previous meta-analysis published in 2020, including 2,218 patients from 15 studies, compared L/Z fluoroscopy method to the conventional strategy for PVI. Consistent with our findings, this meta-analysis also showed a significant reduction in procedural and fluoroscopy time, while complication rates and acute- and long-term success rates did not differ between groups (11). However, the definition of a low fluoroscopy strategy is not well-defined and differed across the enrolled studies.

In our opinion, the ZF approach is characterized by the operator's decision at the beginning of the procedure to pursue fluoroless ablation before inserting catheters, even though radiation may be required later and thus the ZF strategy is deemed unsuccessful. In addition to above, that during PVI most fluoroscopy required at the beginning of the procedure: catheter positioning and transeptal puncture. After the beginning phase operator staff can remove lead aprons and prevent orthopaedic problems. In our analysis, the ZF strategy was achievable in more than 95% of the procedures in the ZF arm.

Z/MF approach is now more extensively used compared to ZF for AF ablation, mainly due to the costs and technical challenges related to systematic use of transoesophageal or ICE for transseptal puncture.

In addition to the technological aspects, operators' experience is also of paramount importance when implementing Z/MF procedures. For obvious reasons, total fluoroless or minimal fluoroscopic PVI procedures have a learning curve of 20–40 cases (33, 34).

Atrial fibrillation may be precipitated secondary factors by hypertension, hyperthyroidism (35), lifestyle factors such as endurance sport (36), smoking (37), cardiomyopathies (38) and channelopathies (39, 40). Considering these factors may prevent future AF attacks regardless of the ablation strategy or may re-evaluate its indication. Thus, further investigation needed.

## Limitations

There are several limitations that should be acknowledged in our analysis. Firstly, we only included 1 randomized controlled trial (RCT), with the majority of data originating from observational studies. However, the lack of heterogeneity in this aspect suggests that the effects of the ZF approach are consistent across different trial designs and are not affected by potential bias. Secondly, significant differences in patient demographics and different modern mapping system including different specific modern tools for AF ablation could have an impact on the results but were not considered in this analysis. The use of a random-effects model helped to mitigate the potential effect of heterogeneity, and the high level of significance supports the validity of our findings. Finally, data on operators' prior experience with the ZF approach for AF ablations were insufficient, which could have an effect on both procedural and safety outcomes.

## Conclusion

In summary, our analysis of 1,593 patients indicates that the ZF approach is a safe and feasible method for patients undergoing catheter ablation for AF. The significant reduction in procedure time and radiation exposure, without compromising the acute and long-term success rates or complication rates, suggest that the ZF approach can be considered as a viable alternative to the NZF approach for AF ablation procedures.

## Data Availability

The original contributions presented in the study are included in the article/**Supplementary Material**, further inquiries can be directed to the corresponding author.
