# Supplementary material for: Zero fluoroscopy catheter ablation for atrial fibrillation: a systematic review and meta-analysis
Source: Front Cardiovasc Med. 2023 Jun 16;10:1178783. doi: 10.3389/fcvm.2023.1178783 (PMC10313423; doi:10.3389/fcvm.2023.1178783)

Table of contents

Supplementary figure 1. Forest plots of total ablation time (Panel A), total fluoroscopy time (Panel B), fluoroscopy dose (Panel C), acute success rate (Panel D) and long term success rate (Panel E). 2

Supplementary Figure 2. Leave-one-out exercise to demonstrate the stability of the effect estimate. Forest plots depict the effects of omitting individual studies on the cumulative effect size of the procedure time (Panel A) and on the risk ratio of complication (Panel B) endpoints in studies comparing zero fluoroscopic vs. non-zero fluoroscopic strategies for atrial fibrillation ablation. 3

Supplementary Figure 3. Analyses of publication bias. Funnel plot analyses of the primary endpoints – procedure time (Panel A) and complications (Panel B) – showed no signals of asymmetry. 4

Supplementary figure 1. Forest plots of total ablation time (Panel A), total fluoroscopy time (Panel B), fluoroscopy dose (Panel C), acute success rate (Panel D) and long term success rate (Panel E).

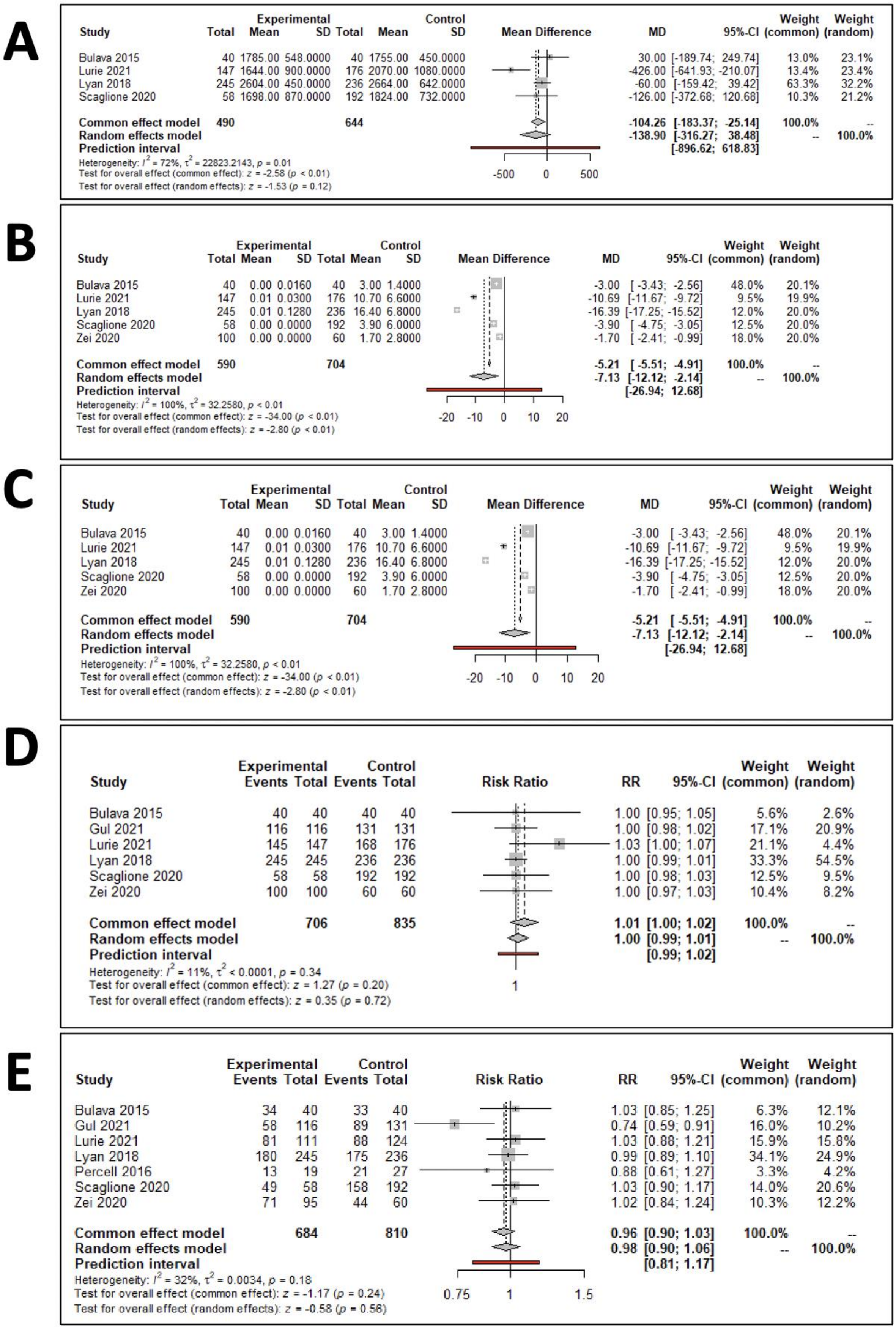

Supplementary Figure 2. Leave-one-out exercise to demonstrate the stability of the effect estimate. Forest plots depict the effects of omitting individual studies on the cumulative effect size of the procedure time (Panel A) and on the risk ratio of complication (Panel B) endpoints in studies comparing zero fluoroscopic vs. non-zero fluoroscopic strategies for atrial fibrillation ablation.

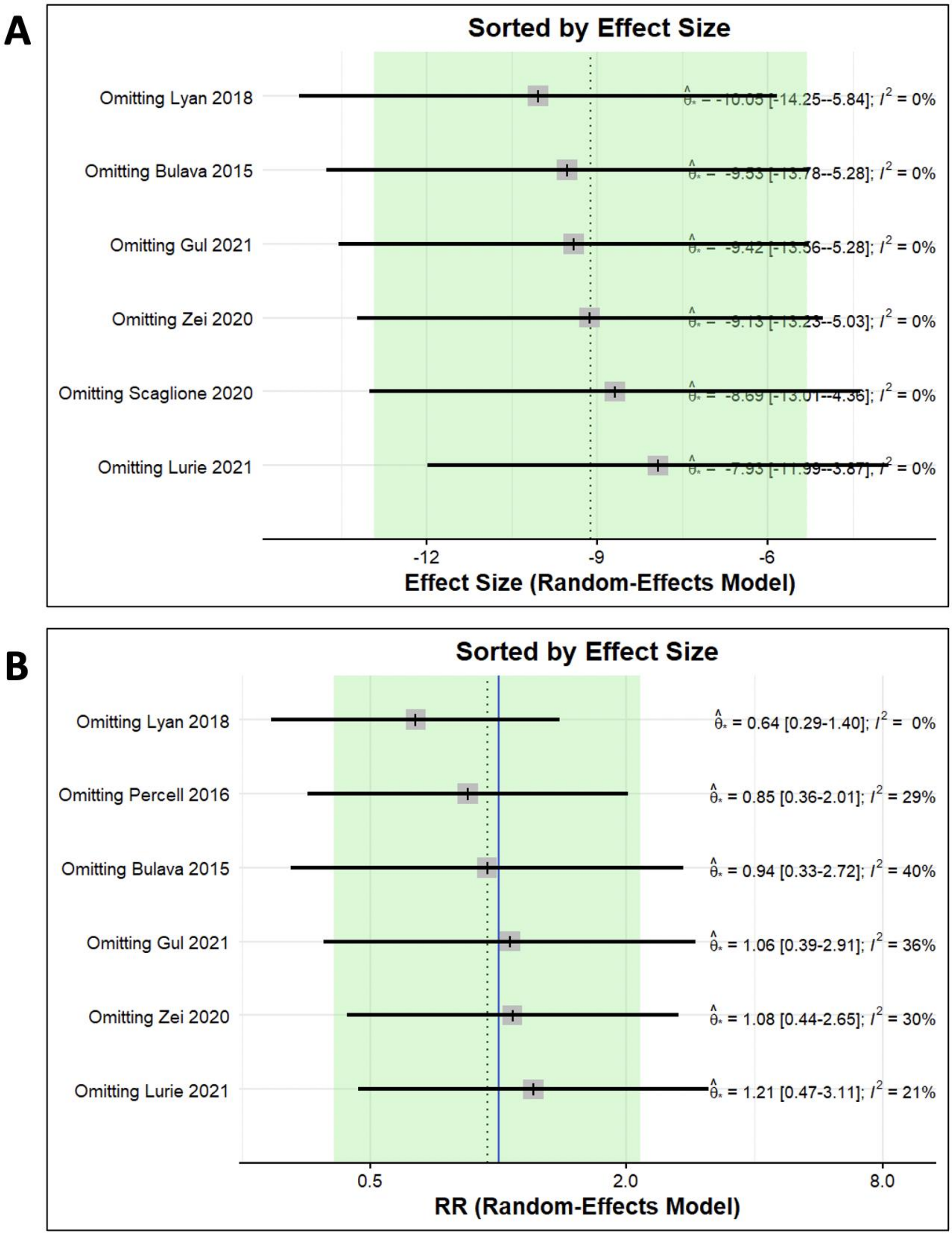

Supplementary Figure 3. Analyses of publication bias. Funnel plot analyses of the primary endpoints – procedure time (Panel A) and complications (Panel B) – showed no signals of asymmetry.

A

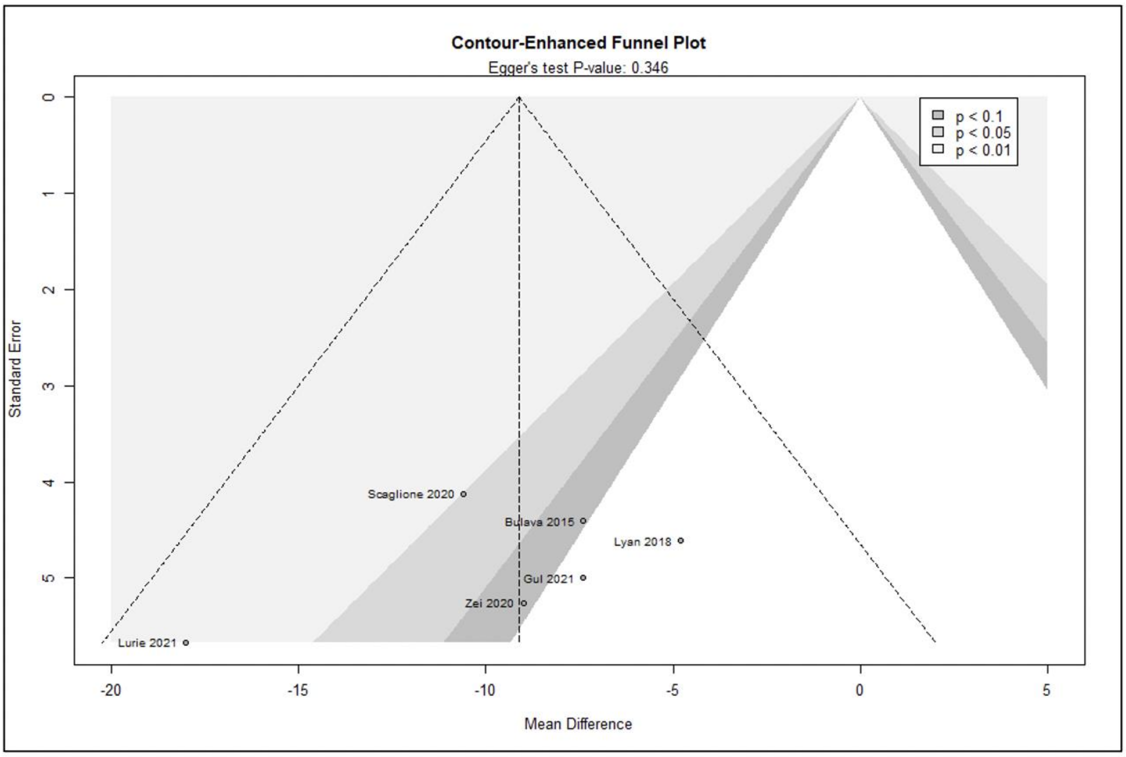

B

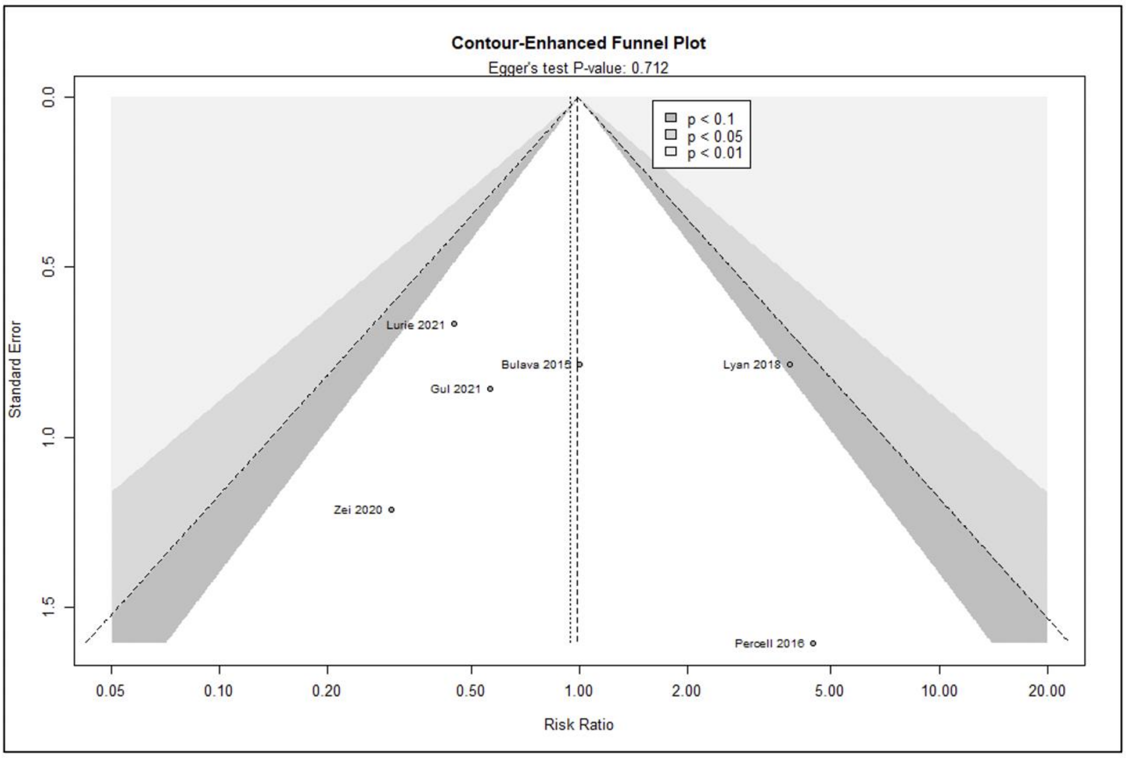

Supplement: Supplementary file 1 [file Datasheet1.pdf]
